# Assessing atypical brain functional connectivity development: An approach based on generative adversarial networks

**DOI:** 10.3389/fnins.2022.1025492

**Published:** 2023-01-09

**Authors:** Pedro Machado Nery Dos Santos, Sérgio Leonardo Mendes, Claudinei Biazoli, Ary Gadelha, Giovanni Abrahão Salum, Euripedes Constantino Miguel, Luis Augusto Rohde, João Ricardo Sato

**Affiliations:** ^1^Center of Mathematics, Computing, and Cognition, Universidade Federal do ABC, São Bernardo do Campo, Santo André, Brazil; ^2^Laboratory of Integrative Neuroscience, Universidade Federal de São Paulo, São Paulo, Brazil; ^3^National Institute of Developmental Psychiatry for Children and Adolescents (CNPq), São Paulo, Brazil; ^4^Department of Psychiatry, Hospital de Clínicas de Porto Alegre, Federal University of Rio Grande do Sul, Porto Alegre, Brazil; ^5^Department of Psychiatry, School of Medicine, University of São Paulo, São Paulo, Brazil; ^6^UniEduK, Jaguariúna, Brazil; ^7^Big Data, Hospital Israelita Albert Einstein, São Paulo, Brazil

**Keywords:** machine learning (ML), biomarker, neural networks, children, functional connectivity, GANs, neurodevelopment

## Abstract

Generative Adversarial Networks (GANs) are promising analytical tools in machine learning applications. Characterizing atypical neurodevelopmental processes might be useful in establishing diagnostic and prognostic biomarkers of psychiatric disorders. In this article, we investigate the potential of GANs models combined with functional connectivity (FC) measures to build a predictive neurotypicality score 3-years after scanning. We used a ROI-to-ROI analysis of resting-state functional magnetic resonance imaging (fMRI) data from a community-based cohort of children and adolescents (377 neurotypical and 126 atypical participants). Models were trained on data from neurotypical participants, capturing their sample variability of FC. The discriminator subnetwork of each GAN model discriminated between the learned neurotypical functional connectivity pattern and atypical or unrelated patterns. Discriminator models were combined in ensembles, improving discrimination performance. Explanations for the model’s predictions are provided using the LIME (Local Interpretable Model-Agnostic) algorithm and local hubs are identified in light of these explanations. Our findings suggest this approach is a promising strategy to build potential biomarkers based on functional connectivity.

## Introduction

Most common and severe mental disorders first manifest during childhood, adolescence, and early adulthood (Kessler et al., 2005; Solmi et al., 2021). Atypical or delayed neurodevelopmental trajectories due to complex interactions between genetic and environmental factors have been proposed as the underlying mechanism for some common mental disorders (Shaw et al., 2007; Di Martino et al., 2014; João Ricardo Sato et al., 2019). Better describing atypical neurodevelopmental trajectories might presumably improve predictions of mental health prognosis and better inform preventive interventions.

More specifically, resting-state fMRI (rs-fMRI) based functional connectivity can be used to assess brain developmental trajectories prospectively and non-invasively in children and adolescents (Grayson and Fair, 2017). Functional connectivity is operationally defined as a measure of statistical dependence between the recorded neural activity of spatially segregated brain regions (Friston, 2011). In a common approach for connectivity analysis, cortical parcellations are used to identify regions of interest (ROIs) presenting homogeneous blood oxygen level-dependent (BOLD) signals (Gordon et al., 2016). ROI-to-ROI pairwise correlations are then used to quantify fMRI signal temporal dependencies yielding the so-called functional connectivity matrix (Li et al., 2009). This data format is an appropriate raw feature set for further analysis, as it summarizes the connectivity states in a given subject and can be collected across subject groups.

Machine learning methods and strategies are increasingly used in clinical research and applications. Among machine learning approaches, artificial neural networks have been extensively adopted due to their good predictive power. In particular, Generative Adversarial Networks (GANs) have shown promising results in the context of semi-supervised learning and generative models, especially in cases in which researchers are interested not only in identifying patterns but also in reliably reproducing them.

Generative adversarial networks (GANs) consist of a double artificial network trained in a game-like environment where two subnets (i.e., generator and discriminator) compete during the optimization process. The generator yields samples from a latent space vector in an attempt to fool the discriminator so that it classifies fake data as real. More generally, the discriminator subnetwork differentiates a modeled pattern from another, helping the generator produce more realistic samples through an adversarial training process.

In this study, we built on these characteristics of GANs to develop and test a normative approach to evaluate atypical trajectories. Models were trained using only neurotypical participants’ functional connectivity matrix data, capturing the variability across participants without a psychiatric diagnosis. For the output scores of these models, neuroatypical functional connectivity patterns should appear as outliers, regardless of the diversity of neuroatypical profiles. Furthermore, this approach allowed us to explore different patterns of neurotypicality by observing the resulting generator subnetwork.

Though still scarce, previous studies have applied GANs and resting-state functional connectivity for data augmentation and illness classification in major depressive disorder (MDD), schizophrenia (SZ) (Zhao et al., 2020), and both attention deficit hyperactivity disorder (ADHD), and autism spectrum disorder (ASD) (Yao and Lu, 2019). Other studies applying GANs to MRI data have been mainly focused on image reconstruction (Shende et al., 2019) and generation (Kazuhiro et al., 2018; Welander and Eklund, 2018; Kwon et al., 2019). In contrast with these previous applications, we used GANs to construct an “atypicality detector” and as a “reproducer” of neurotypical fMRI data patterns.

In other words, it was possible to reproduce the neurotypical state in the generator, while predicting a neurotypicality score in the discriminator using the fMRI dataset collected 3 years before a mental symptoms screening. We expect to differentiate neurotypical real data from both fake generated data and real neuroatypical data. As artificial neural networks have been criticized for interpretability issues, we adopted an explainability algorithm to measure specific large-scale network feature contributions to each model. Furthermore, ensemble strategies were explored to improve classification performance.

## Materials and methods

### Subjects

The data sample employed by this study comprises 550 children and adolescents (53% male) recruited from two public Brazilian schools from São Paulo (site 1, *N* = 274) and Porto Alegre (site 2, *N* = 276) cities. Participants were a subset of the “High Risk Cohort Study for Psychiatric Disorders in Childhood” (HRC, *N* = 2,512 participants, more details in Salum et al., 2015). The age range was between 6 and 15 years old when the fMRI data were acquired, and the Development and Well-Being Assessment (DAWBA) was conducted approximately 3 years later. This assessment allows the classification of subjects in healthy controls and any psychiatric disorder condition, which is interesting for modeling purposes later described. Further cognitive, behavioral, and sociodemographic assessments were performed in the cohort (Salum et al., 2015). Most subjects with psychiatric diagnostics presented emotional disorders, such as major depression, and hyperkinetic disorders such as ADHD. Both sites’ local ethics committees approved the protocol of the study. Written and verbal consent was obtained respectively by the legal guardians and by the children involved in the study.

### Data acquisition protocol

Resting-state fMRI time-courses were obtained with an HDX and an HD 1.5 Tesla Signa MR systems (G.E.), respectively in site 1 and site 2. Both sites acquired scans through a 180 echo-planar imaging (EPI) sequence, with the following acquisition parameters: repetition time (TR) of 2,000 msec, echo time (TE) of 30 msec, flip angle of 80°, slice thickness of 4 mm, gap of 0.5 mm, number of excitations (NEX) of 1, 26 axial slices, 80 × 80 matrix size, 128 × 128, 1.875 × 1.875 mm reconstruction matrix, performing 6 min of acquisition. Subjects remained with eyes open in the scan which contained a fixation point. High-resolution T1 images were acquired for spatial normalization with a three-dimensional fast spoiled gradient recalled echo sequence (TR of 10.91 msec, TE phase of 4.2 msec, flip angle of 15°, thickness of 1.2 mm, field of view of 24.0 × 18.0 cm, NEX of 1 and 256 × 192 matrix size with up to 160 axial slices for whole-brain coverage).

Five hundred and three (503) participants were assessed using the DAWBA 3 years after the fMRI acquisition. The assessment consisted of an interview with parents covering emotional, hyperactivity, and behavioral disorders, as well as more severe disorders (Goodman et al., 2000). Based on this assessment, 377 subjects were considered to be neurotypical (IQ: μ = 102.27, σ = 16.64) whereas 126 were considered neuroatypical (IQ: μ = 100.96, σ = 16.93).

### Preprocessing

Raw fMRI data were preprocessed using the CONN toolbox v.16.b (^1^ Whitfield-Gabrieli and Nieto-Castanon, 2012) based on the SPM12 software.^2^ First, the functional images were unwarped and the head motion was corrected. Then, the brain tissues were segmented into gray and white matter and cerebrospinal fluid. Next, the resultant images were spatially normalized to the MNI ICBM152 template and then functionally normalized to the standard space. After that, data were scanned for outlier detection and scrubbing using the ART approach based on frame displacement > 0.5 mm and global signal z > 3.^3^ Finally, the functional images were spatially smoothed with a full width at a half maximum (FWHM) equal to 8 mm. Linear detrending was performed, as well as bandpass filtering (0.008–0.09 Hz). The nuisance variables (i.e., cerebrospinal fluid and white-matter signals) were regressed out using six head motion parameters (and their respective derivatives), with motion censoring and the Simult approach (Hallquist et al., 2013). The mean frame displacement before motion censoring was 0.23 (std. = 0.29) and post was 0.11 (std. = 0.04).

Local representatives for the 333 regions-of-interest (ROIs) as defined by Gordon et al. (2016) in its functional cortical parcellation were obtained by averaging the voxel’s BOLD signals. This atlas also labels each ROI as belonging to a functional network (RSBFN); Following the previous processing, pairwise temporal correlations were calculated for each pair of different ROIs, resulting in functional connectivity matrices using a Fisher transform. Vectorized upper triangular matrices were used as inputs to the neural networks summing up (333^2^–333)/2 = 55,278 unique features. Using all features for training would be impractical due to the effect of dimensionality on the nets’ optimization algorithm performance, so in the following section we describe a dimensionality reduction procedure.

### Dimensionality reduction

In order to reduce the scope of this study, focusing mostly on cognitive, attentional, and emotional circuitries, which are often linked to neuroatypicality (Krain and Castellanos, 2006), we opted to use up to five predictors in the ensemble construction, one for each of the considered RSBFNs: *Default Mode, Dorsal Attention, Ventral Attention, Fronto-parietal* and *CO* + *SN* [combination of the *Cingulo-opercular* and *Salience* RSBFNs as in Lopez-Larson et al. (2017)] as labeled in the Gordon parcellation atlas.

After the organization of the connectivity matrices in specific functional networks (subsets of features), a cleaning process was carried out. First, a value of −2 was considered the floor and 2 the ceiling, limiting the range of variation. This procedure aims to concentrate the sensitivity of the modeling process within a given significant range of the output of the Fisher transform and facilitate the ingestion in the following steps algorithms. Next, the values were scaled to the interval [−1,1] required by the hyperbolic tangent activation function utilized in the machine learning models. Regarding quality control, an exclusion criterion was applied, and so matrices with more than 15% of modified cells were discarded. This step was conducted to reduce outlier influences.

The above-mentioned cleaning step may cause the exclusion of a subject from one functional network while it is preserved in another, as subsets were processed separately. Thus, the exclusion threshold may result in a variable number of samples for each RSBFN depending on the alterations produced by the cleaning process. In the scenario in which more data were excluded (for the Default, VentralAttn, and CO + SN RSBFNs) this resulted in the loss of 1 unlabeled, 11 neurotypical, and 2 neuroatypical subjects’ data, resulting respectively in 46, 366, and 124 matrices lasting in each group. This effect is later discussed in the ensemble strategies to ensure each predictor gets a vote on samples that were not excluded.

**FIGURE 1:**
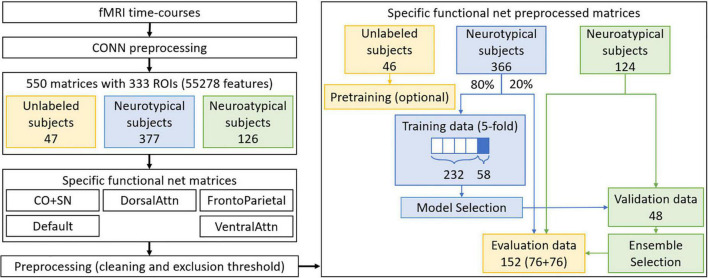
Preprocessing steps performed for datasets preparation. In blue, neurotypical data and processes only involving this type of data are identified. In green, the same is done for neuroatypical data, while parts of the process involving a mixture of these two types of data are identified in yellow. Note that only neurotypical subjects’ data is used for GAN training while a balanced evaluation dataset is used for the evaluation step.

Figure 1 summarizes the final preprocessing steps. From each set of specific functional net preprocessed matrices, only neurotypical subjects’ data are used in training. Approximately 80% of the neurotypical data were used in 5-fold cross-validation, so as to allow variability assessment for each trained model. In the case when the most amount of data exclusion was observed, the greatest multiple of 5 under the 80% limit amounted to 290 matrices. Data from the remaining 76 neurotypical subjects were reserved for the evaluation set, as well as a corresponding number of neuroatypical data samples. The remaining 48 neuroatypical samples and the fold-out data (58 neurotypical matrices, a fifth of the training data, not used in direct optimization of the models) were used respectively for ensemble and model selection, which will be further discussed afterward.

### Generative adversarial networks design and training

**FIGURE 2:**
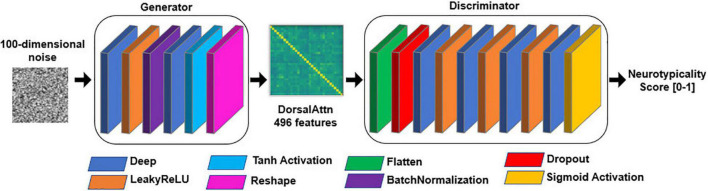
General structure of generative adversarial network (GAN) models. Each layer is only fed forward, represented by the color-coded rectangles. The discriminator subnetwork is fed with batches of both generated (i.e., fake) data from the generator as well as genuine training data. rely on 100-length random noise vectors.

For each specific RSBFN several architectures were trained in a standardized manner and on neurotypical data only. Five-fold cross-validation to assess model stability and variability (especially of the discriminator subnetwork, in terms of the bias-variance tradeoff). Each GAN contained a feedforward structure similar to the one described in Figure 2. A 100-length normally distributed random vector was used as input to all the generator subnetworks.

The output format of the generator was equal to the input format of the discriminator and to the specific number of features for each of the five data sets according to the five RSBFNs used in training. Moreover, the output node of the discriminator is always a single node that outputs a score of neurotypicality for each sample. A variable number of nodes was used in the hidden layers of both generators and discriminators, always constrained to the input and output sizes of each subnetwork.

Due to the variable number of features for each RSBFN (from 276 to 976), we first assessed triangular and rectangular networks with different numbers of neurons at each layer to evaluate stability. GANs are known to have failure modes such as a lack of convergence and mode collapse and they also need to be balanced in terms of their subnetworks (Bhagyashree and Nandi, 2020). Therefore, unbalanced GANs are easy to spot, either because training neuroatypicality scores would not raise for neurotypical training data or because the evolution of these scores was rather noisy. By reducing the number of layers in the generator subnetwork and utilizing Dropout layers in the discriminator network, we were able to achieve a better neighborhood of solutions.

Dropout layers randomly assign a value of 0 to the weights of a given percentage of connections (Abadi et al., 2016). This procedure was applied as a regularization tool stimulating the learning process to be handled without being reliant on a small number of inputs. Also, these layers simulate a smaller number of features being inputted to the first hidden layer in the discriminator, which in the case of bigger sets of features helped to overcome dimensionality issues to some degree. Dropout rates from 40 to 70% were evaluated. Hyperbolic tangent, the Leaky version of the Rectified Linear Unit (LeakyReLU), and Sigmoid activation layers (Abadi et al., 2016) were used in different parts of the GAN models.

A Batch Normalization Layer (Abadi et al., 2016) was utilized in the generator subnetworks. This layer normalizes inputs from the previous layer, applying a transformation that approximates the mean of the output to 0 and its standard deviation to 1. It is able to encompass the learning of scaling parameters during training and acts as a moving average during evaluation/prediction using these parameters. Finally, Flatten and Reshape layers were employed to reshape inputs and outputs into usable formats respectively as tensors and functional connectivity matrices.

Each GAN was trained in the following manner: the discriminator subnetwork was trained in a standalone manner with a mini-batch of 32 real samples. Then, the whole GAN network (considering both subnetworks) was trained using the false information that the generated samples were real in an attempt to trick the frozen discriminator while the generator is optimized. Freezing the discriminator while training the generator keeps the latter from having a decrease in performance allowing an increase in the performance of the former (Abadi et al., 2016). Discriminator and generator losses were calculated using Binary Cross-Entropy considering the above-mentioned desired labels. An Adam optimizer with a learning rate of 0.0002 was utilized in the training process of both nets (Abadi et al., 2016).

Each training process was halted using a stop criterion defined by a training metric. A moving window with 15,000 training epochs was used. Once the standard deviation of the percentage of the training neurotypicality scores above 0.5 went below 0.01 within this window, the training process stopped. The percentage of training scores above 0.5 is an indicator of how sensitive the discriminator has become to the training samples, while the moving standard deviation window for this metric ensures the training will stop when there is little variability in the scores for a specific number of epochs, aiding to avoid overfitting.

### Model and ensemble generative adversarial networks selection

Rectangular and triangular architectures were chosen for each RSBFN based on training stability and performance across training folds: 2 architectures for the Default, CO + SN, and VentralAttn RSBFNs, 3 architectures for the FrontoParietal and 4 for the DorsalAttn network. The performances on the validation and test data sets were not considered for the selection of architectures.

For each architecture, five training folds were performed and evaluated in a fold-out. The model with the best performance on the fold-out (neurotypical) data across folds was chosen to participate in the ensemble construction as the representative of the architechture. The considered performance metrics were the percentage of neurotypicality scores above 0.5 and the mean neurotypicality score for the fold-out matrices.

From individual RSBFN architectures, a combinatorial approach was used to construct ensembles containing 0 or 1 model from each RSBFN, not necessarily containing more than one RSBFN. Besides the null model, this approach generated 539 ensembles.

Ensembles are a technique usually employed with so-called weak predictors that, through bagging or boosting strategies, attempt to improve prediction performance and/or diminish prediction variability (Polikar, 2012). Alternatively, we explored natural divisions in the data set, defined by the five RSBFNs, so predictions are complementary to each other in a more functional perspective. The exclusion of certain RSBFNs aimed to reduce undesired information improving prediction performance and explainability.

For each ensemble, its underlying RSBFNs models were compared in terms of the subjects which were excluded in the preprocessing stage (excessive outlier corrections threshold). If subject A was excluded in the preprocessing of a RSBFN and subject B was excluded in the preprocessing of another RSBFN in the same ensemble, subjects A and B were excluded from the evaluation data. In the case with the most divergence, two matrices relative to neurotypical subjects were excluded due to the impossibility of obtaining reliable predictions for at least one of the models involved.

**FIGURE 3:**
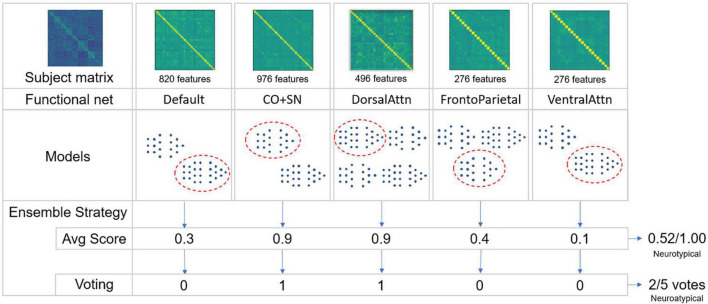
Schematic of the ensemble strategies employed. From a given subject regions of interest (ROI)-to-ROI preprocessed correlation matrix, specific intra-functional net correlations are extracted to reduce dimensionality. For a given number of trained models in each of these functional nets, a single model is chosen for each one of them, generating a score of neurotypicity for its specific functional net. To combinate models’ outputs, two ensemble strategies were applied: Averaging models’ scores and converting these scores to votes to calculate majority. In both cases, not necessarily all five functional nets were used to constitute an ensemble. An exhaustive strategy was used combining each trained model for each functional net with a variable number of functional nets being used. A validation dataset was used to choose the best ensembles.

Two evaluation strategies were implemented. The first assesses the average atypicality score among the ensemble models. The second consisted in a majority voting strategy in which each model in the ensemble assigned one vote defining if a given matrix corresponds to a neurotypical or neuroatypical class (see Figure 3). The mean difference between these two strategies lies in the fact that the magnitude of the neurotypicality score is important in an average score which has to be above 0.5 so that a subject is considered neurotypical. In a voting strategy, all models have an equal contribution–although the same threshold of 0.5 is used to compute the vote across the models. In cases in which an even number of RSBFNs is employed in a voting strategy, a tie will favor a neuroatypical prediction. This approach aims to increase specificity and is coherent with a normative approach, somewhat comparable to anomaly detection.

Combining RSBFN models exhaustively can generate issues related to the randomness of this procedure. Apart from potentially being a misleading factor in the evaluation, it would not provide a single best reliable model. Ranking models based on their performance would only reflect the fact that combining diverging scores may, in some cases, lead to an increase in ensemble performance. Therefore, a validation set consisting of neuroatypical matrices was used to select the best ensembles before effective performance metric quantification was carried out on the balanced evaluation dataset. In cases where the same performance on the validation dataset was observed for two models, the mean fold-out neurotypicality score across the ensemble models was used as a tiebreaker criterion.

Accuracy, negative predictive values (NPV), positive predictive values (PPV), sensitivity, and specificity were obtained from the single best of the implemented ensembles. To evaluate how different from a null model (i.e., random choices) the predictions were, we computed the *p*-value of the balanced accuracy (average between sensitivity and specificity metrics) using bootstrap permutations of the labels in the evaluation dataset. Ideally, a ROC-AUC (area under the receiver operating characteristic curve) metric would be employed, but this metric would not be adequate to evaluate the variation of the threshold of votes (discrete) as well as the threshold for mean scores (continuous). A significance value of α = 0.05 was chosen to test the null hypothesis (i.e., the null model) rejection.

### Explainability of the models

The LIME (Local Interpretable Model Agnostic) algorithm (Ribeiro et al., 2016) was employed to improve the explainability of the individual GAN discriminator models. This technique performs a local linear approximation for a given prediction, computing the importance of features for that sample, without necessarily including all features as important to this prediction (Ribeiro et al., 2016). This is useful when identifying which features in each RSBFN have the most weight to the predictions, that is, which connections are considered more important for the assessment of neurotypicality (i.e., possible local hubs with important dysconnectivity effects).

As each feature corresponds to a connectivity measure between two ROIs, it is possible to take an average of all importance values for each feature across the evaluation data to identify connections that were determinant to the model predictions. Likewise, one can average all connection importance values across a given ROI for the matrix resulting from the previous step, quantifying ROI centrality in the RSBFN based on prediction values.

Higher mean values indicate greater importance for a given functional connection to the considered class (i.e., neurotypical or neuroatypical). From that, we can infer which relations hold most of the importance for the prediction of neurotypicality. Similarly, ROI centrality as described here shows the importance of a given ROI as a local hub within the specific RSBFN in which the prediction was made.

For the above-mentioned procedure, we chose the trained models with the highest individual (considered in ensembles with only themselves) accuracy for each of the five RSBFNs used and obtained a LIME explanation for all samples in the evaluation dataset. ROI centrality was considered separately for neurotypical and neuroatypical groups.

## Results

**FIGURE 4:**
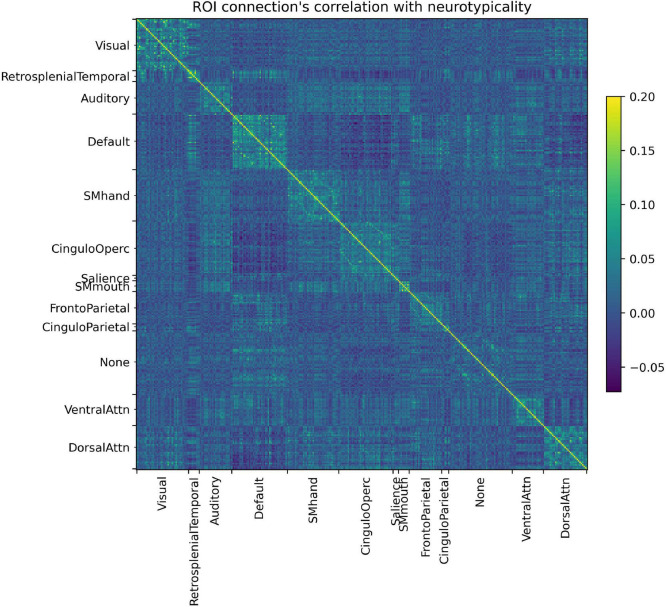
Regions of interest (ROI) connections’ correlation with neurotypicality label. The complex pattern observed justifies the use of a machine learning model, as no direct associations can be made directly from data.

In Figure 4 it is possible to observe how each ROI connection is correlated with the neurotypicality label. Not only do several connections show little to no correlation with neurotypicality, but also intricate patterns are observed in the connections that do. In Figure 4, features are in fact only in areas around the main diagonal (intra-RSBFN relations).

**FIGURE 5:**
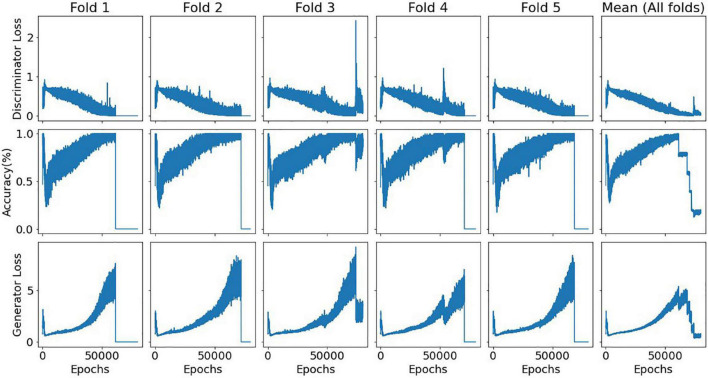
Evolution of the discriminator accuracy and both discriminator and generator losses for a single DorsalAttn model with a triangular architecture. Relative consistency across folds is displayed, showing model stability, especially in terms of the bias-variance tradeoff for the discriminator subnetwork. In all folds except the third, it is noticeable when the training process was halted due to the predefined stop criterion. A seed number of 0 was used for this specific training process (set for both NumPy and Tensorflow modules).

An estimated 45-min time frame was observed for each training fold using a machine with an i7-9750H processor with 2,6 GHz–with Python Tensorflow module v.1.15.0, Keras module v.2.2.4 and NumPy v.1.19.2. The workload was mostly memory and CPU-bound. Figure 5 illustrates the evolution of the discriminator accuracy (genuine vs generated samples discrimination) and both discriminator and generator losses for the single Dorsal Attention model. This specific model contained 200 nodes in its generator single hidden layer, 50% of dropout rate, and sequential 75, 50, and 25 nodes in its discriminator hidden layers.

**FIGURE 6:**
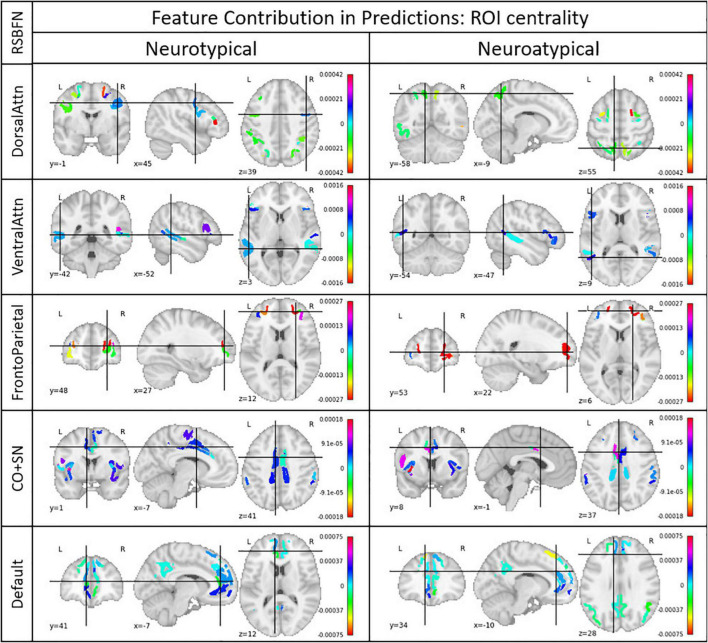
Regions of interest (ROI) centrality. Each ROI belonged to a Resting State Brain Functional Network (RSBFN) and only intra-RSBFN relations were considered. The referred models presented the highest accuracy among the architectures tested for their specific datasets. Centrality (color bar) was derived from the average connection importance in predictions across neurotypical and neuroatypical subjects. Positive values indicate ROIs whose connections raised the neurotypicality score while negative values indicate ROIs whose connections lowered this metric. High values (in module) indicate great importance for the neurotypicality score. Group separation aims to ensure that the group’s neurological differences are excluded as confounding factors.

Mean and voting evaluation strategies are equivalent and most of the models ranked low in the validation data set performance. Accuracies varied between 0.51 and 0.59, with similar values for balanced accuracy. The best individual models in terms of accuracy were used for the computation of ROI centrality described in subsection “Model and ensemble GANs selection.” In Figure 6 it is possible to observe these results for each of the five studied RSBFNs for both neurotypical and neuroatypical subjects.

Considering models grouped in ensembles, first we can consider the use of the highest fold-out percentage scores above 0.5 as the fold selection method. Using the average neurotypicality score across ensemble models as an evaluation metric, we obtain a summary metric. The results for the best model in this category as defined by the validation data criterion for ten seeds (random parameter initialization of GAN weights) are shown in Table 1.

**TABLE 1:** Ensemble performance of models with a mean score as evaluation metric and the highest percentage of fold-out scores above 0.5 for fold selection.

Method	Mean score									
Seed	0	1	2	3	4	5	6	7	8	9
Accuracy	0.54	0.53	0.51	0.54	0.59	0.55	0.55	0.59	0.57	0.57
Sensitivity	0.71	0.61	0.76	0.74	0.70	0.67	0.68	0.78	0.43	0.57
Specificity	0.37	0.46	0.25	0.34	0.47	0.43	0.42	0.41	0.70	0.57
PPV	0.53	0.53	0.50	0.53	0.57	0.54	0.54	0.57	0.59	0.57
NPV	0.56	0.54	0.51	0.57	0.61	0.57	0.57	0.65	0.55	0.57
Balanced accuracy	0.54	0.53	0.51	0.54	0.59	0.55	0.55	0.59	0.57	0.57
*p*-value	0.19	0.24	0.44	0.21	0.03	0.09	0.12	0.01	0.05	0.05

Note that the performance variables and *p*-values are seed-dependent. In this case, four of the ten different seeds yielded a model providing typical/atypical diagnosis prediction better than chance (i.e., balanced accuracy > 0.5).

Models with one and three predictors were among the highest-ranking ones for the aforementioned strategy. Although, in general, they presented high *p*-values for the metric of balanced accuracy which did not allow for null hypothesis rejection. The accuracy was greatly affected by the performance in the neurotypical class, which is noticeable from specificity values. This suggests that an average neurotypicality score across models may be sensitive to high individual scores (i.e., favoring the predictions of neurotypical scores–above 0.5).

Voting strategies for the same fold selection method showed improved performance when compared to the previous evaluation strategy, as shown in Table 2. The best voting strategy outcomes were seen for ensembles considering two predictors, which can be explained by the number of votes required to assign a neuroatypical prediction in these cases. If both models in an ensemble with two predictors vote for a neurotypical phenotype the final prediction does so. The divergence between models can be exploited as an anomaly detector–as the neuroatypical class was not modeled, individuals in this class should appear as outliers with low neurotypicality scores. Voting strategies also address the sensitivity of averages to outliers (faulty scores in the ensemble who weigh in strongly for a neurotypicality prediction), diminishing the negative effect that models in different training points could have when combined.

**TABLE 2:** Ensemble performance, with votes as evaluation metric and the highest percentage of fold-out scores above 0.5 for fold selection.

Method	Vote									
Seed	0	1	2	3	4	5	6	7	8	9
Accuracy	0.49	0.53	0.61	0.56	0.60	0.55	0.59	0.55	0.59	0.57
Sensitivity	0.53	0.39	0.75	0.47	0.58	0.53	0.61	0.66	0.39	0.51
Specificity	0.46	0.66	0.47	0.64	0.62	0.57	0.57	0.45	0.78	0.63
PPV	0.49	0.54	0.59	0.57	0.60	0.55	0.58	0.54	0.64	0.58
NPV	0.49	0.52	0.65	0.55	0.59	0.54	0.59	0.57	0.56	0.56
Balanced accuracy	0.49	0.53	0.61	0.56	0.60	0.55	0.59	0.55	0.59	0.57
*p*-value	0.70	0.27	< 0.01	0.10	0.01	0.17	0.02	0.14	0.01	0.04

Note that 5 of the 10 different seeds resulted in models providing typical/atypical diagnosis prediction better than chance (i.e., balanced accuracy > 0.5).

Fold selection strategies showed little to no difference in the result of the above-mentioned evaluation strategies. The highest mean neurotypicality score from fold-out often chose the same fold of the former strategy (i.e., based on the percentage of scores above 0.5).

## Discussion

In this study, we employed a normative approach to model neurotypicality from functional connectivity profiles. We used a GAN model trained on functional connectivity matrices from neurotypical subjects only, having been able to replicate this pattern to new samples in its generator and providing a neurotypicality score in its discriminator. As our main result, this proposed score is promising regarding the prediction of children’s mental health 3 years in advance. Moreover, we proposed and tested some model training, selection, ensemble, and evaluation strategies.

First, we observed that model performance is dependent on parameter initialization, i.e., it depends on the seed used in the random initialization of the GAN weights. Most studies in the literature rely on the results and performance from a single trained GAN model, without assessing the impact of the seeds used for network weights initialization. Second, the vote strategy (Table 2) as an evaluation metric to build the neurotypicality scores provided slightly better performance in the test data, with balanced accuracy of almost 60%. Third, based on the LIME approach for model explanation, we explored ROI centrality to identify local hubs inside RSBFNs with potential dysconnectivity effects (Figure 6).

In addition, we observed that single model performance varied significantly, even within each RSBFN. This may be due to the aggressive Dropout layer used to reduce the number of trainable parameters. Given the high dimensionality of functional connectivity variables (ROI-to-ROI), the aggressive dropout was relevant to eliminate irrelevant features improving the models’ robustness.

Previously in the wide literature, GANs have displayed their potential in image generation tasks (Radford et al., 2015; Salimans et al., 2016). Aside from phenotypic characterization, at least two previous studies generated connectivity matrices that have been used for data augmentation (Yao and Lu, 2019; Zhao et al., 2020) aiming to alleviate the low sample count effect and improve classification performance. Though not directly comparable to this study–due to the use of a discriminative approach and larger datasets in both cases–these attempts highlight how effective GANs can be for neurological data handling. Discrimination tasks involving ASD and ADHD achieved 90.2 and 87.9% accuracy in relation to matched healthy subjects, respectively (Yao and Lu, 2019). In the other study, MDD and SZ discrimination tasks (against healthy controls) achieved 70.1 and 80.7% accuracy, respectively (Zhao et al., 2020). Previous results suggest that generated data may hold at least part of the predictive value compared to that which would be observed with real samples–it could be further exploring characteristics of the underlying real data distribution at the cost of some redundancy.

In the last decade, connectome analysis serves as a framework upon which neurodevelopmental trajectories can be assessed *in vivo* (Di Martino et al., 2014; Grayson and Fair, 2017). In the scope of the current study, only intra-RSBFNs correlations were explored but further models could be trained considering all combinations of RSBFNs (visible in Figure 4), which would characterize inter- RSBFNs interaction. Moreover, in the current study, the generation of five separate RSBFN typical patterns still does not comprehensively consider the whole brain typicality. Thus, we believe the models’ performance could be improved. Future studies could focus on further compressing features in a reversible form to address both dimensionality issues as well as the whole brain atypical connectome generation.

## Conclusion

We conclude that GAN is a promising tool for anomaly detection and in the context of brain functional networks, it could be used to build a functional atypicality score. In the current study, we illustrate the proposed approach in a proof-of-concept that this score could predict psychiatric problems 3 years in advance with accuracy greater than chance. Methods for accuracy optimization are still an open question.

In the long run, methods as proposed in this study may support clinical procedures by helping both diagnosis and prognosis. Models’ specificity and sensitivity values provide a starting point on how clinicians should evaluate the neurotypical score. In addition, the model explainer can provide reason behind each of these inputs, and the evaluation can be done using the resting-state fMRI alone regardless of preexisting suspicion for neuroatypicality. For a more comprehensive and collective view of what features were considered most important for models predictions, Figure 6 characterizes relevant hubs from a functional perspective, though their centrality from other points of view is rather complex neurobiologically. We prefer not to provide a detailed explanation of the role of each region-of-interest in the psychiatric disorders, since given the dataset used, these would be mostly conjectures. It is important to mention that in this study, we focused on proposing a new approach and illustrating its value in a proof-of-concept application.

## Data availability statement

The original contributions presented in the study are publicly available. This data can be found here: https://osf.io/ktz5h/wiki/home/.

## Ethics statement

The studies involving human participants were reviewed and approved by School of Medicine–University of São Paulo Federal University of São Paulo Federal University of Rio Grande do Sul. Written informed consent to participate in this study was provided by the participants’ legal guardian/next of kin.

## Author contributions

PD conducted the analyses and wrote the manuscript. SM reviewed the manuscript and provided support in the analyses. CB reviewed the manuscript and provided support in the results interpretation. AG collected the data and reviewed the manuscript. GS, EM, and LAR coordinated data collection and reviewed the manuscript. JS provided support in data analysis, wrote, and reviewed the manuscript. All authors contributed to the article and approved the submitted version.

## Footnotes

1
https://www.nitrc.org/projects/conn/

2
http://www.fil.ion.ucl.ac.uk/spm/software/spm12/

3
https://www.nitrc.org/projects/artifact_detect/
